# Single-cell transcriptomic profiling for inferring tumor origin and mechanisms of therapeutic resistance

**DOI:** 10.1038/s41698-022-00314-3

**Published:** 2022-10-10

**Authors:** Maoxuan Lin, Moshe Sade-Feldman, Lori Wirth, Michael S. Lawrence, Daniel L. Faden

**Affiliations:** 1 Department of Otolaryngology-Head and Neck Surgery, Massachusetts Eye and Ear, Boston, MA 02118 USA; 2 Massachusetts General Hospital Cancer Center, Boston, MA 02118 USA; 3 Broad Institute of MIT and Harvard, Cambridge, MA 02142 USA; 4 Harvard Medical School, Boston, MA 02115 USA; 5 Department of Medicine, Massachusetts General Hospital, Boston, MA 02118 USA

**Keywords:** Molecular medicine, Translational research, Cancer genomics, Oral cancer

## Abstract

Head and Neck Squamous Cell Carcinoma (HNSCC) is an aggressive epithelial cancer with poor overall response rates to checkpoint inhibitor therapy (CPI) despite CPI being the recommended treatment for recurrent or metastatic HNSCC. Mechanisms of resistance to CPI in HNSCC are poorly understood. To identify drivers of response and resistance to CPI in a unique patient who was believed to have developed three separate HNSCCs, we performed single-cell RNA-seq (scRNA-seq) profiling of two responding lesions and one progressive lesion that developed during CPI. Our results not only suggest interferon-induced APOBEC3-mediated acquired resistance as a mechanism of CPI resistance in the progressing lesion but further, that the lesion in question was actually a metastasis as opposed to a new primary tumor, highlighting the immense power of scRNA-seq as a clinical tool for inferring tumor origin and mechanisms of therapeutic resistance.

## Introduction

Head and neck squamous cell carcinoma (HNSCC) will account for an estimated 54,000 new cases and 11,230 deaths in the United States in 2022^1^. Despite advances in therapy, the prognosis of HNSCC remains poor with a 5-year survival of 67.4%, due to local and distant recurrences and the development of second primary cancers^2–5^. The development of multiple primary tumors is most commonly ascribed to field cancerization from tobacco exposure^6^ or from multisite infection from Human Papillomavirus (HPV) in HPV-associated HNSCC^7–9^. Differentiating a recurrence from a second primary cancer can be challenging and is based on clinical features such as anatomic location and time course, as opposed to genomic features. Importantly, treatment for a recurrent HNSCC differs considerably from that of a new primary tumor.

Anti-PD-1 checkpoint inhibitors (CPI) are now standard-of-care for recurrent or metastatic HNSCC^10,11^. However, most patients do not have a durable response to CPI^12^. Mechanisms of resistance to CPI in HNSCC remain poorly understood^13^. Most studies of CPI resistance focus on the immune microenvironment, particularly the deactivation and exhaustion of T and B cells. Biomarkers for understanding resistance to immunotherapy, including PD-L1, TIM3, LAG3, VISTA, GIFR, and TIGIT, have been identified in different cancers, including HNSCCs^14–19^. There has been less focus on tumor cell-intrinsic mechanisms of immunotherapy resistance in HNSCC. Recent studies in other tumor types, including work from our group, suggest that apolipoprotein B mRNA-editing enzyme, catalytic polypeptide-like 3 (APOBEC3) family members may be a mechanism of immune evasion^20–24^. Importantly, APOBEC is known to be active in HNSCC and is a significant source of DNA alterations^25–27^.

To identify drivers of response and resistance to CPI in a unique patient who was believed to have developed three separate HNSCCs, we performed single-cell RNA-seq (scRNA-seq) profiling of two responding (partial response, PR) lesions and one non-responding (progressive disease, PD) lesion that developed during CPI. Our results not only suggest interferon-induced APOBEC3-mediated acquired resistance as a mechanism of immunotherapy resistance in the progressing lesion but further, that the lesion in question was actually a metastasis as opposed to a new primary tumor, highlighting the immense power of scRNA-seq as a clinical tool for inferring tumor origin and mechanisms of resistance to therapy.

### Case

**Fig. 1:**
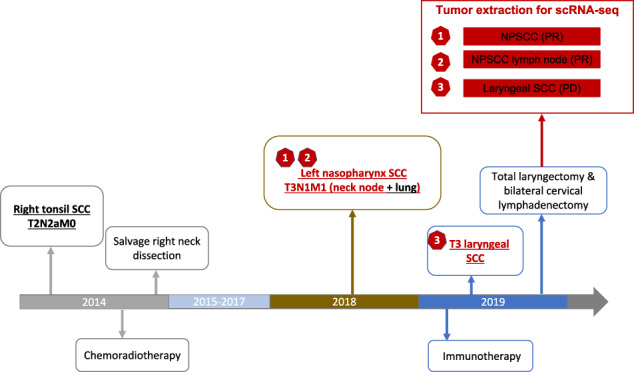
Timeline of cancer progression and treatment. The tumors investigated are colored in red.

The patient is a 50-year-old male smoker who was diagnosed with a squamous cell carcinoma (SCC) on the right tonsil in 2014 for which he received definitive chemoradiotherapy and subsequently, a salvage right cervical lymphadenectomy (Fig. 1). Four years later the patient developed a second cancer, this time, a left nasopharyngeal SCC (NPSCC; Tumor 1) with metastases to the left neck (Tumor 2) and the lung. Due to the lung metastasis, he was treated with the CPI nivolumab. The lung metastasis had a complete response and the primary NPSCC and neck lymph node metastases had partial responses which remained stable over time. The patient then presented with a new laryngeal mass which developed while on CPI, confirmed by biopsy to be SCC (Tumor 3). Because this cancer had a location in the aerodigestive tract remote from the existing cancer which had responded to CPI, and the classic appearance and anatomic positioning for a mucosally-based SCC arising from the vocal cord, it was presumed to represent a third primary malignancy. This cancer was therefore treated with a total laryngectomy and bilateral cervical lymphadenectomy.

## Results

**Fig. 2:**
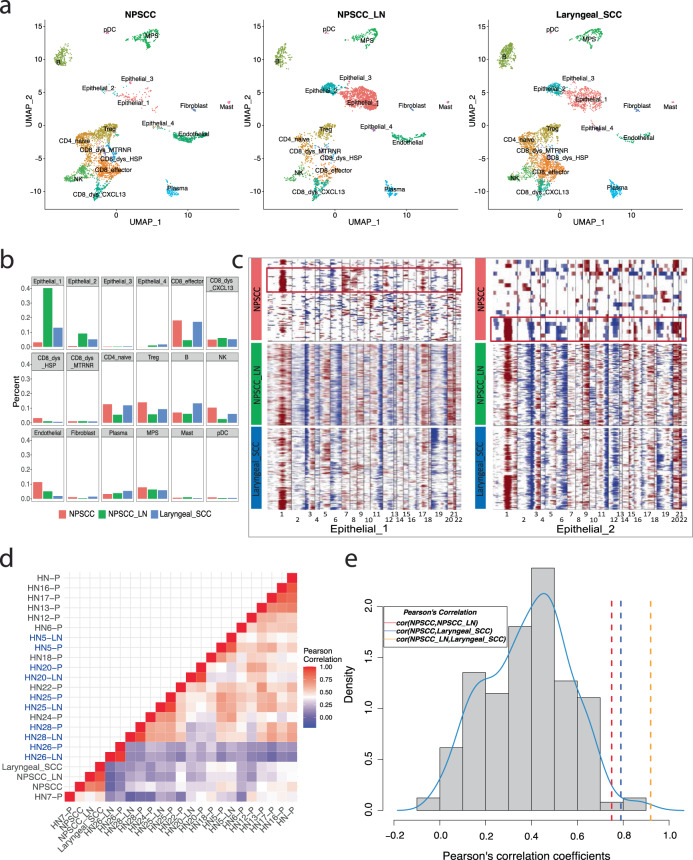
Copy number alteration profiles suggest the newly developed laryngeal SCC is a metastasis from the NPSCC, as opposed to a new primary tumor. **a** UMAP plots and **b** bar graphs show different cell compositions among three tumors. Dysfunctional CD8+ T cells were split into three subtypes by their marker genes: CD8_dys_CXCL13 with marker gene CXCL13, CD8_dys_HSP with heat shock proteins, and CD8_dys_MTRNR with mitochondrially encoded RNA genes. NPSCC and NPSCC lymph node have distinct components of malignant cells especially Epithelail_1 and Epithelial_2 clusters, demonstrating the inter-tumor heterogeneity. **c** Heatmap of copy number alteration profiles inferred from two main malignant cell clusters of three tumors using T cell and B cell as “normal” references. Dark red indicates genomic amplifications and blue indicates deletions. The x-axis shows all autosomes in numerical order. The y-axis displays epithelial cells from different tumors (left: Epithelial_1; right: Epithelial_2). Nearly identical copy number alterations observed between the NPSCC lymph node and the laryngeal SCC are similar to those of subclones of the NPSCC tumor (red box). **d** Heatmap of Pearson’s correlation matrix of 23 HNSCC tumors’ CNA profiles. Correlation coefficients were calculated using mean CNA levels of tumors inferred from scRNA-seq using inferCNV. Five matched pairs of primary tumors (-P) and lymph node metastases (-LN) are in blue font. Matched pairs and three HNSCC tumors investigated in this study show higher similarity within the pairs (red squares), than between unpaired tumors. **e** Histograms of Pearson’s correlation coefficients of non-paired HNSCC tumors’ CNA profiles, excluding the five matched pairs and three tumors investigated in this study. Three tumors investigated here show higher similarity (*r* = 0.75, 0.79, and 0.92) than that would be expected randomly, with one-sided *p*-values of 0.023, 0.013, and 0.0017, respectively.

To understand the heterogeneity in the patient response to CPI treatment, we performed scRNA-seq on the three isolated lesions (all extracted at the same time): Tumor 1—NPSCC (PR), Tumor 2—NPSCC lymph node (PR), and Tumor 3—laryngeal SCC (PD). After the removal of cells that did not pass QC (see “Methods”), a total of 11,470 cells remained for downstream analyses (NPSCC: *n* = 3818, NPSCC lymph node: *n* = 2870, laryngeal SCC: *n* = 4782), representing 18 different cell clusters (Fig. 2a, b, Supplemental Fig. 1A, B). The three specimens showed differing cell compositions (Fig. 2a, b, Supplemental Fig. 1C, D) with the NPSCC having the largest percentages of Treg and NK cells and the smallest percentage of epithelial cells, the laryngeal SCC having the largest number of B cells, and the NPSCC lymph node dominated by epithelial cells. Copy number alterations (CNAs), using T cells and B cells as reference “normal” cells, were used to confirm the malignant nature of epithelial cells (Fig. 2c). While copy number heterogeneity was observed in three tumors and different malignant cell clusters with distinct gene expression profiles (Fig. 2a, c), surprisingly, the NPSCC lymph node and the newly developed laryngeal SCC demonstrated nearly identical copy number alterations (Pearson’s correlation coefficient *r* = 0.92; Fig. 2d, e), including typical HNSCC altered regions such as gain of chromosome 3q and loss of 3p, as well as gain of 1q and loss of 11q, which were also observed in subclones of the primary NPSCC tumor (*r* = 0.75, 0.79 between NPSCC and NPSCC lymph node, laryngeal SCC, respectively). CNA analyses of 20 additional HNSCC’s scRNA-seq from Puram et al.^28^ showed that five matched pairs of primary tumors and lymph node metastases, have highly concordant CNA profiles, similar to the three lesions in this study, which is not observed in non-paired tumors (Supplemental Fig. 2, Fig. 2d). The probability of observing a CNA similarity of *r* >= 0.75 between any two random HNSCC was found to be extremely small (one-sided *p*-value = 0.023; Fig. 2e). Taken together, these analyses strongly suggest that the laryngeal SCC is a metastasis from the NPSCC as opposed to a new primary tumor.

**Fig. 3:**
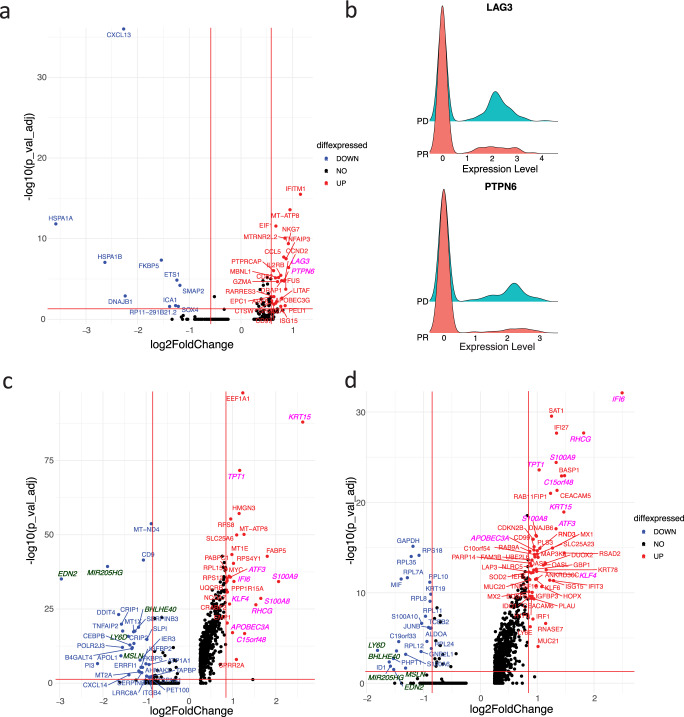
Increased expression of T cell exhaustion markers LAG3 and PTPN6, APOBEC3A, and interferon-inducible protein IFI6 in the progressing laryngeal SCC. **a** Volcano plot of differentially expressed genes in dysfunctional CD8+ T cell (CD8_dys_CXCL13) in laryngeal SCC vs NPSCC and NPSCC lymph node shows increased expression of two T cell exhaustion markers LAG3 and PTPN6 (italicized pink font) in laryngeal SCC. Only genes with log2FoldChange larger than 0.25 were shown. Wilcoxon Rank Sum tests were performed and *p* values were adjusted by Bonferroni correction. Horizontal red line: −log10(0.05); vertical red lines: +/−log2(1.5). **b** Ridge plots of expression of LAG3 and PTPN6 in tumors with fold changes of 1.80, 1.79 and adjusted *p* values of 1.3e−5, 1.8e−5, respectively, comparing the laryngeal SCC (PD) to two regressing lesions (PR). **c**, **d** Volcano plots of differentially expressed genes in Epithelial_1(C) and Epithelial_2(D) in laryngeal SCC vs NPSCC and NPSCC lymph node. Commonly upregulated and down-regulated genes in laryngeal SCC were shown in italicized pink and dark green fonts, respectively, supporting increased APOBEC3A expression stimulated by interferon signaling. Horizontal red line: −log10(0.05); vertical red lines: +/−log2(1.8).

To identify mechanisms of resistance employed by the metastatic lesion (laryngeal SCC) which developed and progressed on CPI, the two regressing lesions were first combined to increase the statistical power (Fig. 2a) and then compared with the progressing lesion for differential gene expression analyses. Expression of immune-checkpoint factors in T-cell immunity, such as programmed death-1 (PD-1) and cytotoxic T-lymphocyte-associated protein 4 (CTLA-4) are critical in mediating response to CPI^13^. Of common T-cell exhaustion markers, LAG3 and PTPN6 were observed to be differentially expressed in the progressing laryngeal SCC, compared to the regressing lesions (Fig. 3a, b), suggesting the emergence of new inhibitory receptors in exhausted CD8+ T cells as one mechanism of CPI resistance.

**Fig. 4:**
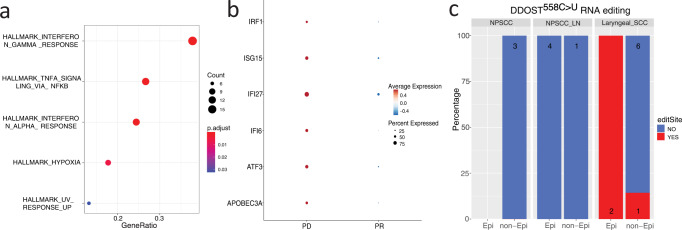
Upregulated interferon-related genes and elevated APOBEC3A activity in Epithelial 2 cluster in the newly developed laryngeal tumor suggest an interferon-induced APOBEC3A upregulation driving acquired resistance to CPI. **a** Gene set enrichment analysis of differentially expressed genes (absolute fold change >1.8 and adjusted *P* value <0.05) in Epithelial 2 cluster in laryngeal SCC vs two regressing lesions. **b** Dot plot of increased expression of APOBEC3A, ATF3, IFI6, IFI27, ISG15, and IRF1 in Epithelial 2 cluster in laryngeal SCC (PD) vs two regressing lesions (PR). **c** DDOST^558C>U^ RNA editing is exclusively observed in laryngeal SCC, mainly in tumor cells. Epi: epithelial cell; non-Epi: non-epithelial cells.

For malignant cells, we examined differentially expressed genes between the two largest malignant cell clusters in the progressing laryngeal SCC compared to the regressing nasopharyngeal tumors (Fig. 3c, d). Of the top 10 commonly upregulated genes in the laryngeal SCC, APOBEC3A was found to have fold changes of 2, 1.9 and adjusted *p* values of 9.4e−18, 1.9e−15, in the Epithelial 1 and Epithelial 2 clusters, respectively. An interferon-induced gene, interferon alpha inducible protein 6 (IFI6) also showed higher expression in the laryngeal SCC with fold changes of 2.04, 5.6, and adjusted *p* values of 5.2e−35, 7.8e−33 in the Epithelial 1 and Epithelial 2 clusters, respectively. Additional interferon-related genes such as IRF1, ISG15, and IFI27 were also upregulated in the Epithelial 2 cluster (Fig. 3d). Interferon signaling pathways were the most enriched signatures in gene set enrichment analysis of differentially expressed genes in the Epithelial 2 cluster (Fig. 4a). The co-upregulation of multiple interferon-related genes in the Epithelial 2 cluster (Figs. 3d and 4b) suggests increased APOBEC3A expression stimulated by interferon signaling. Increased APOBEC3A activity was further validated by the detection of DDOST^558C>U^ RNA editing exclusively in the laryngeal SCC (Fig. 4c, Supplemental Fig. 3). Together, these findings support an interferon-induced upregulation of APOBEC3A as a mechanism of CPI resistance in the progressing Laryngeal SCC.

## Discussion

In this n-of-1 study, we demonstrate the potential clinical utility of scRNA-Seq for inferring tumor origin and mechanisms of therapeutic resistance. We performed single-cell transcriptomic profiling of three HNSCC lesions from the same patient, two of which responded to CPI and the third which developed de novo while the patient was receiving CPI. Clinically, the newly developed lesion was felt to be a new primary based on the clinical context, anatomic location, and appearance; however, in depth copy number alteration profiling suggests this was in fact a new metastasis from the existing NPSCC. Chromosomal abnormalities are widespread in human cancers and have been used previously to determine tumor origin. Worsham et al. reported common clonal origin of two synchronous primary HNSCC originating from separate sites based on shared aneuploidy patterns^29^. Recently, Puram et al. demonstrated concordant copy number alteration profiles between five matched primary HNSCC and lymph node metastases inferred from scRNA-seq, with validation of CNA profiles and their similarity using whole exome sequencing^28^. This finding holds important clinical implications as the patient was treated surgically for this presumed new cancer, while would have been treated non-surgically, if it was known to be a metastasis, suggesting that the resolution provided by scRNA-Seq in this setting, could have altered clinical care.

We further identified increased expression of T cell exhaustion markers *LAG3* and *PTPN6*, as well as interferon-related genes and elevated *APOBEC3A* activity in this recurrence, suggesting a combination of immune-regulated and tumor cell-intrinsic APOBEC3A-mediated resistance to CPI in the progressing lesion. APOBEC-mediated mutagenesis is pervasive in multiple tumor types, such as bladder, cervical, breast, lung cancers, and particularly, HNSCC^24–27,30–32^. APOBEC mutagenesis has been reported to be involved in the acquisition of driver mutations^24,33–35^ and tumor subclone diversification^35–37^. Multiple studies have suggested the critical role of APOBEC3A upregulation in therapeutic resistance^21,22^. Law et al. showed that APOBEC-mediated therapeutic resistance develops while on therapy and suggest that the mechanism is likely C-to-U editing coupled with normal DNA repair process leading to DNA mutations (21). Our group demonstrated that certain therapeutics can induce APOBEC3A expression, leading to sustained mutagenesis in drug-tolerant cancer cells^20^. In addition, CRISPR-mediated deletion of APOBEC3A significantly reduced double-strand DNA breaks and delayed the emergence of drug resistance, indicating APOBEC3A as a potential target for preventing or delaying acquired resistance in lung cancer therapy^20^. Interestingly, others have found that APOBEC3A expression induces cellular PD-L1 and cell-surface PD-L1 expression^23^. APOBEC3A-induced PD-L1 upregulation requires its deaminase activity and suggests APOBEC3A as an immune evasion mechanism and a potential target for improving cancer therapeutic efficacy of PD-1/PD-L1 targeting therapy. While induction of PD-L1 by APOBEC3A would suggest a mechanism of immune evasion and resistance, such an effect should theoretically lead to increased sensitivity to CPI, which was not seen in this patient’s resistant tumor.

Interferons (IFNs) are considered the main modulators of the APOBEC3 family expression^25,38^. For instance, IFN-α can induce APOBEC3A expression through IFN-sensitive response elements in the putative promoter region of APOBEC3A^39^. While IFN-α itself was not differentially expressed in the PD laryngeal SCC, IFI6 and IFI27, two type I IFN-α stimulating genes, showed significantly higher expression in this new tumor. IFI6 and IFI27 have been reported to be highly expressed in HNSCC compared to normal tissues^40^ and have been shown to promote tumor development and metastatic potential of multiple cancers^41,42^. The detailed mechanisms of IFI6 and IFI27 regulating tumor growth however have not been well elucidated. Our finding of their co-upregulation with APOBEC3A in the newly developed tumor under CPI suggests they may promote tumor progression via upregulating APOBEC3A activity, further inducing APOBEC3A mutagenesis. Taken together, co-expression of high levels of APOBEC3A, IFI6, and IFI27 in the laryngeal SCC suggests that interferon-induced APOBEC3A upregulation could be a potential driver of therapeutic resistance to CPI in HNSCC. Increasing evidence combined with existing knowledge of the role APOBEC plays in mutagenesis in multiple cancer types strongly suggests APOBEC3A as a mediator of tumor response to therapy across multiple tumor types.

In summary, our findings support an interferon-induced APOBEC3A-mediated resistance to CPI in HNSCC, in addition to the deactivation and exhaustion of T cells. This study also demonstrates the power of scRNA sequencing in determining the origin of tumors, which has direct implications for treatment decisions. As this study lacks in vitro and in vivo studies to confirm the computational findings from scRNA-seq, future studies are needed to validate and further explore the role of APOBEC3A in HNSCC therapeutic resistance. Confirmation of the role of APOBEC-mediated resistance could help refine patient selection for enhanced therapeutic efficiency and approaches to overcoming CPI resistance.

## Methods

Written informed consent to a protocol approved by the Dana-Farber/Harvard Cancer Center and Boston University Institutional Review Board was obtained prior to sample collection. The study was conducted in accordance with the U.S. Common Rule. Clinical Response was determined by RECIST Criteria Version 1.1.

We utilized single-cell RNA sequencing with the VDJ NextGEM v1.1 10x Genomics Chromium platform to profile three freshly isolated tumor samples collected at the time of total laryngectomy: (1) the nasopharyngeal tumor (PR), (2) the nasopharyngeal cancer lymph node metastasis (PR), and (3) the new laryngeal cancer (PD). Preprocessing of the single-cell RNA-seq data was done using the Cell Ranger pipeline provided by 10x Genomics. Quality control was performed with the following metrics: (1) doublet detection and removal by Scrublet^43^; (2) cell-level metric: number of genes >= 300 & <= 3000, unique molecular identifier (UMI) counts >= 500, mitochondrial ratio <0.2, and average expression of housekeeping genes >0.8, compiled based on Tirosh et al.^44^, where gene expression was normalized by log((raw_count_per_gene)/(total_count_per_cell)*10,000 + 1)); (3) gene-level metric: genes detected in <10 cells were excluded. Downstream analyses were performed using the Seurat package v4.0.3^45–48^. Specifically, integrative analysis was applied to cluster together the same cell types across the three tumors, followed by the identification of marker genes for each cluster, and cell types were then manually annotated based on these marker genes (Supplemental Fig. 1A, B). Malignant cells were identified by copy number alterations inferred using inferCNV^49^, and Pearson’s correlation coefficients between tumors’ mean CNA levels were calculated to quantify their similarity. CNA analyses were also performed on 20 additional HNSCC’s scRNA-seq data from^28^, including 5 marched tumor-lymph node metastasis pairs, to get the distribution of similarity between paired and non-paired HNSCC’s CNAs as a reference for calculating the probabilities of observing similarity in the three tumors investigated here. Differential gene expression analyses were performed between the progressing lesion and regressing lesions using the Wilcoxon Rank Sum test and *p* values were adjusted by Bonferroni correction. Molecular Signatures Databse (MSigDB) hallmark gene sets^50,51^ provided in R package msigdbr (v7.4.1) were used for gene set enrichment analysis ran by clusterProfiler(v3.18.1)^52^.

### Reporting summary

Further information on research design is available in the Nature Research Reporting Summary linked to this article.

## Supplementary information

REPORTING SUMMARY

Supplemental Material

## Data Availability

scRNA-Seq data files are available in GEO, accession number GSE213047.
