# Supplementary material for: Single-cell transcriptomic profiling for inferring tumor origin and mechanisms of therapeutic resistance
Source: NPJ Precis Oncol. 2022 Oct 10;6:71. doi: 10.1038/s41698-022-00314-3 (PMC9548500; doi:10.1038/s41698-022-00314-3)
Supplement: Supplementary file 2 — Supplemental Material [file 41698_2022_314_MOESM2_ESM.docx]

**Supplemental Figure 1**. Cell clustering and cell compositions. (A) UMAP colored by cell-type annotation. (B) Heatmap of the most differentially expressed marker genes for each cluster. (C,D) Cell compositions between three tumors for immune cells (C) and epithelial cells (D).

**Supplemental Figure 2**. Heatmap of copy number alteration profiles of 20 HNSCC tumors from Puram et al ^28^ inferred by inferCNV using T cell and B cell as “normal” references. Dark red indicates genomic amplifications and blue indicates deletions. The x-axis shows all autosomes in numerical order. Five matched pairs of primary tumors and lymph node metastases (HN5, HN20, HN25, HN26, and HN28) show highly similar CNA profiles.

**Supplemental Figure 3**. Increased DDOST^558C>U^ RNA editing activity in the newly developed laryngeal SCC. Aligned sequencing reads are shown for the NPSCC (top), NPSCC lymph node (middle), and laryngeal SCC (bottom) from the patient. The DDOST^558C>U^ RNA editing hotspots (chr1:20981977) are shown in the center, surrounded by the dashed lines. Reads with RNA editing activity are colored in green (A, with its TpC site on the antigenomic strand), while reads without RNA editing activity are completely gray (reference sequence), indicating the increased RNA editing activity in the newly developed laryngeal SCC.
